# *Lutzomyia longipalpis* urbanisation and control

**DOI:** 10.1590/0074-02760150207

**Published:** 2015-11

**Authors:** Oscar Daniel Salomón, María Dora Feliciangeli, María Gabriela Quintana, Margarete Martins dos Santos Afonso, Elizabeth Ferreira Rangel

**Affiliations:** 1 Instituto Nacional de Medicina Tropical, Puerto Iguazú, Misiones, Argentina; 2 Consejo Nacional de Investigaciones Científicas y Técnicas, Argentina; 3 Universidad de Carabobo, Facultad de Ciencias de la Salud, Centro Nacional de Referencia de Flebótomos y Otros Vectores, Maracay, Venezuela; 4 Universidad Nacional de Tucumán, Instituto Superior de Entomología, San Miguel de Tucumán, Argentina; 5 Fundação Oswaldo Cruz, Instituto Oswaldo Cruz, Laboratório de Transmissores de Leishmanioses, Rio de Janeiro, RJ, Brasil

**Keywords:** *Lutzomyia longipalpis*, American visceral leishmaniasis, urbanisation, control

## Abstract

Since the description of *Lutzomyia longipalpis* by Lutz and Neiva more than 100 years ago, much has been written in the scientific literature about this phlebotomine species. Soares and Turco (2003) and Lainson and Rangel (2005) have written extensive reviews focused on vector-host-parasite interactions and American visceral leishmaniasis ecology. However, during the last two decades, the success of *Lu. longipalpis* in colonising urban environments and its simultaneous geographical spreading have led to new theoretical and operational questions. Therefore, this review updates the general information about this species and notes the more challenging topics regarding the new scenario of urbanisation-spreading and its control in America. Here, we summarise the literature on these issues and the remaining unsolved questions, which pose recommendations for operational research.

## Current American visceral leishmaniasis (AVL) situation and *Lutzomyia longipalpis*

At the beginning of the century, new risk factors favouring a greater man-vector contact emerged worldwide, leading to new epidemiological scenarios and the increasing incidence of leishmaniases (Desjeux 2001). Due to socioeconomic factors, urbanisation has been the most relevant epidemiological change for AVL. The massive exodus of infected dogs from rural to urban areas led to a disordered increase of poor sanitary conditions where*Lu. longipalpis* successfully established itself and spread. In Brazil, since the first report in the cities of Teresina, state of Piauí, and São Luís, state of Maranhão (MA) (Arias et al. 1996), AVL has spread from the Northeast to the Central-West, North and Southeast, with cases in at least 25 cities and in 19 out of the 27 states (Maia-Elkhoury et al. 2008, MS/SVS/DVE 2014). This situation has raised a major concern and serious challenges (Costa 2008, Oliveira et al. 2008). Therefore, it is thought that in this new epidemiological scenario, changes in the control program are necessary (Dantas-Torres & Brandão-Filho 2006).

This same trend of AVL spreading with urbanisation has been observed in Paraguay since 1998, when the first infected dogs (Canese 1998) and the first two human cases were detected in Asunción (Canese 2003). AVL extended to neighbouring areas, leading to at least 130 cases in 2010 (Canese 2010), with cases also in the Brazil-Paraguay and Argentina-Paraguay border (Cousiño 2006).

In Argentina, the first record of urban *Lu. longipalpis* occurred on the border with Paraguay, close to Asunción (Salomón & Orellano 2005). After the emergency of the disease in the city of Posadas in 2006 (Salomón et al. 2008), the vector spread to the south along with cases of canine AVL in the provinces of Misiones, Corrientes, Entre Ríos and Formosa, rising to 107 registered human cases of AVL in 2012 (Santini et al. 2013). The vector was recently reported in Uruguay (Salomón et al. 2012) and the province of Salta, Argentina, close to the Bolivian border (Bravo et al. 2013).

The successful control of AVL remain as a great challenge given the context of environmental changes, in which the unknown nature of impacts on the local ecosystems plus the complexities of the new realities of an urban scene suggest new questions to be discussed. Urbanisation of the most important vector, *Lu. longipalpis*, is currently the biggest obstacle for control programs (PAHO 2011, 2013).

A different situation has occurred in Venezuela and Colombia. In Venezuela, the first urban AVL cases were reported in 1998 in a suburb of the city Valencia, in the north-central area of the country (Aguilar et al. 1998). Afterwards, only sporadic cases have been registered in that focus and in other continental cities in endemic areas of transmission, as it had been in the past. However, a sudden outbreak was registered in 2004-2005 in suburbs of the city Porlamar and in new settlements in Margarita Island, an ancient focus of AVL (Pifano & Romero 1964). This outbreak was related to a massive immigration from rural areas and from abroad. An intensive effort of the AVL Control Programme of the Ministry of Health of Venezuela aimed for a prompt diagnosis and early treatment, vector and reservoir control and health education and was able to control the situation. This outcome was favoured by the fact that the problem was circumscribed to an island. In this focus, *Lu. longipalpis* and *Lutzomyia evansi* are sympatric, but so far only *Lu. longipalpis* is considered to be the vector as it has been found naturally infected by *Leishmania infantum* that is indistinguishable from that isolated from humans and dogs (Feliciangeli et al. 1988, Rodríguez et al. 2005). However, the role of *Lu. evansi*in the transmission of AVL in the island is not to be discarded. In fact, both sandflies have been found to be infected in other foci (Feliciangeli et al. 1999).

In Colombia, *Lu. longipalpis* has been reported in rural areas in the Magdalena River Valley in the departments of Santander, Cundinamarca and Tolima y Huila and in the Caribbean Coast in the departments of Córdoba and Sucre y Bolívar, where it is sympatric with *Lu. evansi*. In 1997 in Guatiguará, Piedecuesta (Santander), a massive migration of poor families occurred and a rural settlement was established. Between 1998-2000, eight cases of AVL were reported. In a long-term study (May 1999-May 2000), natural infection with the subgenus*Leishmania* spp was found in 1.93% of cases (Flórez et al. 2006). In suburbs of Sincelejo (Bejarano et al. 2001) and El Carmen de Bolivar (Cortes & Fernández 2008),*Lu. longipalpis* seems thus far to not be present and*Lu. evansi* is regarded as the only possible vector. It can therefore be concluded that there is not a significant increase in the incidence of AVL in the urban areas of Venezuela and Colombia and that AVL is occurring mainly in rural areas. To our knowledge, there have been no reports of urban AVL in other Latin American countries (PAHO 2011).

## The urbanisation-dispersion of *Lu. longipalpis*

A great deal of information about *Lu. longipalpis*-AVL was already reviewed (Soares & Turco 2003, Lainson & Rangel 2005) *.*Therefore, we here aim to discuss the new information generated after these reviews to focus on the urbanisation-dispersion of *Lu. longipalpis*, which involves climatic, environmental and sociocultural factors. So far, joined phenomena of the urban colonisation of the vector and spread of AVL were attributed to the confluential change in the biology of the vector adapting from sylvatic to domestic environments, environmental changes such as deforestation and human migration, parasite spill over from sylvatic reservoirs and dispersion by infected dogs (Lainson & Rangel 2005, Costa 2008, Rangel & Vilela 2008, Maroli et al. 2013).

The urban emergence is reported as: (i) "first" AVL autochthonous urban or atypical cases (Duarte et al. 1994, Noyes et al. 1997, Carrillo et al. 1999, de Lima et al. 2009, de Campos et al. 2013, Silva et al. 2014a, Von Zuben et al. 2014), (ii) urban outbreaks (Aguilar et al. 1998, Delgado et al. 1998,Feliciangeli et al. 1999, Bevilacqua et al. 2001, Zerpa et al. 2002, Flórez et al. 2006, de Oliveria et al. 2006, Mestre & Fontes 2007, Salomón et al. 2008, Barata et al. 2013), (iii) the finding of *Lu. longipalpis* in urban environments or areas without previous records (Paula et al. 2008,Souza & Borges 2008, Andrade Filho & Brazil 2009, Souza et al. 2009, Salomón et al. 2011a, b, Valderrama et al. 2011, Brazil et al. 2012, Santos et al. 2012, Acosta et al. 2013, Bravo et al. 2013) and (iv) predicted changes of distribution or altitudinal shifts (González et al. 2014). On the other hand, the literature recorded the spread of the vector and infected reservoirs/hosts by *L. infantum* in urban environments from the Northwest to the Southeast of Brazil (Maia-Elkhoury et al. 2008, Alves 2009), reaching Paraguay in the year 2000 (Cousiño 2006), Argentina in 2004 (Salomón & Orellano 2005) and Uruguay in 2010 (Salomón et al. 2011a), increasing the risk of urban AVL in the Southern Cone of South America (PAHO 2011).

When AVL emergence or *Lu. longipalpis* spread are discussed, the actual evidence from recent events should be taken into account, such as the lack of previous knowledge of cases or the presence of vector. New reports could appear due to the increase in the awareness of AVL, acquired availability of diagnosis tools at the local level and "filling the gap" designs between two known endemic areas. In addition, some cases require in-depth studies to confirm or discard autochthony (Martín-Sánchez et al. 2004). Furthermore, "urbanisation" is the behaviour change to be adapted to highly modified urban environments and "dispersion" is the colonisation of areas outside the known geographical range of the species; these are different concepts with particular characteristics at different scales in time and space (Quintana et al. 2012). In the case of *Lu. longipalpis*, the spread was actually recorded as the dispersion of the urbanisation. Therefore, we will prefer to discuss the urbanisation-dispersion issue at each scale.

*Macro-scale* - At this scale, the dispersion was associated with macro-economic trends in the exchange of goods (roadway construction), land use and the integrated energy supply networks that involved massive migrations of populations with their pets from endemic areas, the progressive deforestation (from "fishbone" to crop-extensive cultures), changes in the value of the land and thus unplanned urban growth in poor socioeconomic conditions (new housing developments at rural-urban fringes) and likely local climate changes due to regional human interventions (dam building, irrigation systems, urban heat islands). The AVL in the municipality of Rio de Janeiro was related to deforestation in a reserve to install high-voltage power lines (Marzochi et al. 2009), in the states of São Paulo (SP) and Mato Grosso do Sul (MS) with the construction of the Marechal Rondon Highway and the Brazil-Bolivia gas pipeline (Correa-Antonialli et al. 2007, Cardim et al. 2013, de Almeida et al. 2013) and in Mato Grosso with migration flows (Mestre & Fontes 2007). The following sequential reports of AVL spread from MS (de Oliveira et al. 2000) to Paraguay (Cousiño 2006) and to Argentina and Uruguay (Salomón & Orellano 2005, Salomón et al. 2008, 2011a, 2011a, b, Gould et al. 2013) was not related to any particular event; however, the same "macro" drivers as those for the intensified exchange of goods (even dogs of specific breeds), environment modification and unplanned urbanisation affected the whole region. In this sense, the dispersion of the vector should be distinguished from that of canine AVL, as in Argentina infected dogs were in the whole territory despite the absence of the vector due to dog management in the form of transit and traffic, breeding, adoption and training practices (Salomón et al. 2012).

The sequential records of *Lu. longipalpis* from Campo Grande (MS), to Salto, Uruguay, in 10 years (Figure), in places without records of *Lu. longipalpis* in previous studies, suggest a dispersion of 1,255 lineal kilometres, following ecological conditions of high suitability for the vector (de Almeida et al. 2013, de Andrade et al. 2014). The pheromone of the males of *Lu. longipalpis* from Campo Grande, Asunción and Posadas were characterised as (*S*)-9-methylgermacrene-B (Bray et al. 2009, Brazil et al. 2009, Salomón et al. 2010). In SP, the (*S*)-9-methylgermacrene/*Lu. longipalpis* populations were associated with the spread to the west, while the sibling cembrene-1/*Lu. longipalpis* populations were restricted to places with historical rural records (Casanova et al. 2015). Therefore, when the urban adapted-spreading populations reach a new area, the contiguity of appropriate landscapes and climate might have favoured dispersion, while climate trends and commercially privileged routes also contributed to defining the "least-cost paths" (Fischer et al. 2011). In this regard, the intermittent colonisation of *Lu. longipalpis* at the borders of its current distribution seems to be associated with critical climatic factors (Szelag et al. 2014) and according to potential distribution models, the variables that best generalised the models of the presence of *Lu. longipalpis* in Argentina were the rainfall during the driest quarter and the mean temperature during the coldest quarter (MG Quintana, unpublished observations), the temperature seasonality and annual mean precipitation in MS (de Almeida et al. 2013) and the semiarid and hot climates of the older rural foci of Brazil (Nieto et al. 2006). On the other hand, the southernmost populations of *Lu. longipalpis* could belong to a different subspecies of the species complex, so the hypothesis of the "urbanisation" of previous rural populations with very low-recorded abundance (Salomón et al. 2008, 2010) could be proposed. However, the progressive phenomena at the macro-scale showed a trend in space and time that supports a common "dispersion of urbanisation" event from Brazil to Uruguay, even with genetic flux of the invasive and pre-existent subspecies.

**Figure:**
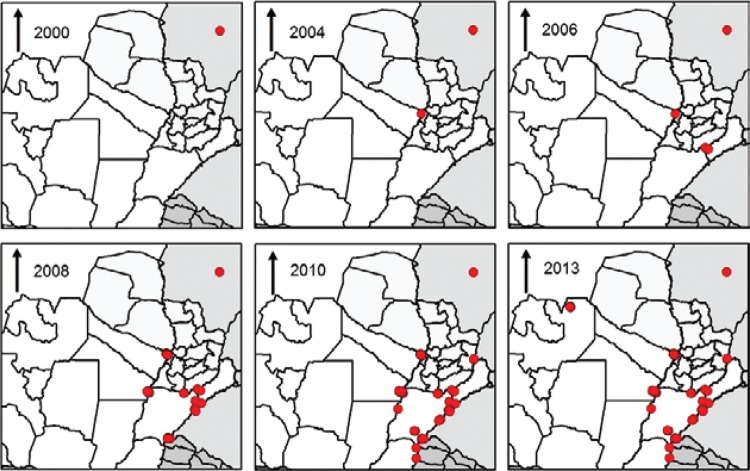
First report (red dots) of *Lutzomyia longipalpis* by year in the South Cone area of the Americas.

*Meso-scale* - Once *Lu. longipalpis* colonises a city, the distribution within the heterogeneous urban landscape is on the meso-scale, usually showing a pattern of "hot-spots" of high abundance, areas of low abundance and areas without vector (patches or "islands" of presence in a sea of absence). The criteria used to define high abundance in the literature is usually an operational one, as natural breaks or quartiles, because there are no data about estimated thresholds of transmission, especially as this could also differ between foci according to the supply of parasites and availability of hosts. In Posadas, the changes in the vector distribution between 2007 (3 human AVL cases) and 2009 (24 cumulated AVL cases) showed limited growth, expansion and change of position for the sandfly patches of abundance, a pattern consistent with metapopulation dynamics where the high-abundance hot spots could act as source populations (Fernández et al. 2013). Further, in Clorinda, where urban *Lu. longipalpis* were reported for the first time in Argentina, the high abundance sites were persistent from 2004-2014 and the new ones may come to extinction as sink populations (Salomón et al. 2009). This persistent clustering was also simultaneously observed in Dracena, SP (Rangel et al. 2012, Holcman et al. 2013).

Many studies have tried to explain the distribution of *Lu. longipalpis* at this scale by association with environmental variables.*Lu. longipalpis* abundance was associated with indexes such as normalised difference vegetation index (NDVI) and normalised difference water index with extreme values (de Andrade et al. 2014) or did not show a clear correlation (Saraiva et al. 2011), but the scales between the vector abundance (spatial buffer of trap attractiveness) and the pixels used to compute the indexes are never consistent. In rural settlements of Venezuela, the proximity to the woodland was correlated with *Lutzomyia pseudolongipalpis* abundance and AVL risk (Feliciangeli et al. 2006) and in urban areas; green patches within highly urbanised areas were associated with the initial steps of *Lu. longipalpis* colonisation (Brazil 2013) during the process toward its final urbanisation (Carvalho et al. 2010, Colla-Jacques et al. 2010, Nascimento et al 2013a). In cities with high landscape heterogeneity and green areas mixed with human dwellings on almost every block, for this variable it is only critical to define a broad suitable area for the vector, excluding just the central downtown and the external rural-periurban ring (Santini et al. 2012); there, intermediate meso/micro-scales (macro-habitat, see micro-scale below) could better explain the distribution.

*Lu. longipalpis* is more abundant in peridomiciles, even in rural environments (Ferreira et al. 2013, Pinheiro et al. 2013, Rêgo et al. 2014) or in transitional secondary forest-recent deforested areas (Pinto et al. 2012, Carvalho et al. 2013a), although the presence of forest edges close to residences was suggested as a potential shelter during the use of insecticides (Oliveira et al. 2012). However, bias in the actual distribution pattern of the population due to trapping designs or transitional events should be taken into account, as the absence of blood sources close to the traps could yield low capture rates in sylvatic environments (Oliveira et al. 2012, de Campos et al. 2013, Carvalho et al. 2013b, Rodrigues et al. 2013) and so produce higher capture rates in recent human settlements with clustered blood sources (Vilela et al. 2011, Paula et al. 2013) or when urbanisation is ongoing simultaneously with the destruction of the original sylvatic habitats (Lainson & Rangel 2005, Marzochi et al. 2009, Alves et al. 2012, Queiroz et al. 2012).

For the abiotic variables associated with vector distribution in the meso-scale*(* Michalsky et al. 2009a), as in the macro-scale, the critical variable or its value could differ between areas and seasons (i.e., rainfall is critical in a dry area, but not in a subtropical area without a dry season or the minimal temperature is critical only in winter) or between sibling species populations. This kind of relativism of critical variables may explain many contradictory data in the literature. Therefore, although the correlation of abundance with climatic variables is still very important to understand the dynamics of risk at each site, it is very difficult to be extrapolated as a species characteristic, where one can conceptually miss both the biological plasticity of the species, the biotic-abiotic contextual scenarios and the climatic variability needed to explain the disparate results from different geographical areas.

*Micro-scale* - In highly micro-heterogeneous environments such as urban landscapes, micro-scale analysis allows for the discrimination of spatial buffers of increasing radius, from the microhabitat - the site of trapping and the area of attractiveness of the trap device-to macro-habitats - from 100-500 m, the autocorrelation distance (Fernández et al. 2013). Furthermore, at this scale the presence of *Lu. longipalpis* could be discriminated into distribution models from the abundance. With this approach, the presence of the vector was associated with macro-habitat variables (tree and bush cover at 100 m, vegetation cover and NDVI at 200 m), while the abundance was better associated with variables at both the micro and macro-habitat levels, such as peridomiciles with accumulated unused material and greater number of plan-pots and number of tree species (de Oliveira et al. 2012, Santini et al. 2012, Fernández et al. 2013). Therefore, the habitat characteristics that promote reproductive success are also associated with AVL prevalence (Belo et al. 2013a, b).

The presence of domestic animals is usually associated with *Lu. longipalpis* abundance (Alves et al. 2012, Queiroz et al. 2012, Costa et al. 2013). In many studies, chickens are the most frequent blood meal source detected in fed females of *Lu. longipalpis* (Alexander et al. 2002, de Oliveira et al. 2008,Missawa et al. 2008, Sant'Anna et al. 2008, Afonso et al. 2012, Soares et al. 2013), secondary to dogs. The chickens could act as a sandfly population amplifier and breed site provider and create a micro-climate shelter inside chicken coops (Feliciangeli 2004,Oliveira et al. 2012, Casanova et al. 2013). The breeding microhabitat could also be related to the differential distribution of bacterial colonies in the soil (Peterkova-Koci et al. 2012). However, the abundance of sandflies at this scale could also be driven and biased by the host availability and density close to the trap device, as will be discussed below, in addition to vector preference. For instance, armadillo was the main blood source in rural areas and humans in periurban settings (Macedo-Silva et al. 2014).

An important consideration for control strategies is that the ratio between intradomestic and peridomestic captures varies between sites and seasons. Thus,*Lu. longipalpis* was reported with higher indoor collections (Almeida et al. 2010, Dias et al. 2011, Alves et al. 2012, Silva et al. 2014b), higher peridomestic collections, consistent throughout the years (Michalsky et al. 2009b, Oliveira et al. 2012, Holcman et al. 2013) and even equally distributed captures between peridomicile and intradomicile (Nascimento et al. 2013b). The dog seems to be preferred to man even when the human is located 5 m from the dog and interposed between sandfly populations and dog plume odour (Santini et al. 2010), so the captures could be biased by dogs sleeping indoors/outdoors (de Andrade et al. 2014).

Despite the attractiveness of the dog, no direct statistical association is usually found between the spatial distributions of *Lu. longipalpis*presence/abundance and AVL in dogs or humans (Margonari et al. 2006, Missawa & Dias 2007, Michalsky et al. 2009b, Salomón et al. 2009, Barata et al. 2013, Silva et al. 2014b, Spada et al. 2014). This result may be due to inconsistencies between the scales of sandfly trapping and surveillance accumulated data or because the AVL distribution is more related to cultural and social practices (i.e., networks of dog breeding) rather than to the actual sites with vectorial transmission (Salomón et al. 2012).

The mini-light traps are among the most standardised trapping device used, but as no animal bait increases the attractiveness as in Shannon or Disney traps, they only captured sandflies attracted to light, usually from 2-6 m in proximity and compete with other light sources such as the moon or urban brightness (Davies et al. 1995, Galati et al. 1997, Alexander 2000, Gaglio et al. 2014).

*Active/passive dispersion* - Capture-recapture experiments in rural environments in Colombia showed that 49% of the recaptured *Lu. longipalpis* were found between 0-50 m from the release site, 48% within 100 m and 300 m and nearly 3% at 0.5 km or more (Morrison et al. 1993). In Campo Grande, the dispersal recorded was up to 165 m and 241 m for males and females, respectively, although 92.4% were recaptured at the release site (de Oliveira et al. 2013). From a meso-scale perspective, as was mentioned, the spatial auto-correlation of *Lu. longipalpis* abundance in Posadas, was 500-600 m (Fernández et al. 2013) and from a macro-scale perspective, the literature shows by allelic frequencies that hundreds of individuals per generation migrate between a peridomestic habitat and a 1.2-km distant undisturbed landscape (Márquez et al. 2001). Therefore, we can think at the micro-scale about the probability of an active dispersion by adults or larvae and at meso-scale about the active spread of adults according to a metapopulation dynamics "step by step", including the passive dispersion of pre-imaginal stages by soil or plants with soil and of adults in containers or by the wind. However, at the macro-scale, the dispersion is difficult to conceptualise because of the limited flight range, the static nature of*Lu. longipalpis* populations and the improbability of unintentional transportation by man, arguments used by Lainson and Rangel (2005) to support the hypothesis of an American origin of *L. infantum*. However, as was mentioned in a previous section, the spreading capacity varies between *Lu. longipalpis* sibling populations discriminated by the male pheromone (Casanova et al. 2015), so much data should be reviewed. Nevertheless, the issue of active/passive dispersion remains unexplained despite the fact that it is essential to develop predictive maps at each scale, informing control strategies and proper resource allocation.

## Control

One of the more exhaustive reviews on the attempts and methods used to control sandfly vectors of visceral leishmaniasis and cutaneous leishmaniasis in the Old and New World was by Alexander and Maroli (2003).

More recently, in a wider context addressing AVL control, intervention trials against vectors combined with interventions against reservoirs (dog collars, culling, vaccination) or human treatment have been reviewed by Romero and Boelaert (2010). The results and opinions on the measures used according to focal situations were discussed and were sometimes difficult their interpretation due to difficulty with attributing the results to one or another interventions (Costa et al. 2007).

The activities aimed to vector control depend upon the AVL transmission characteristics at each locality (MS/SVS/DVE 2014). For the development of such actions, to determine the exact site of transmission is essential as it may note a domestic transmission detected by the presence of *Lu. longipalpis* in the environment. In this sense, an effective strategy for reducing disease would be vector control, especially in dwellings and peridomestic environments.

In this review, we have gathered methods and results that were obtained only through studies aiming to control the population abundance of *Lu. longipalpis* to reduce the man-vector contact. We do not include works on the use of dog-collars as this method is actually intended to reduce canine visceral leishmaniasis, reducing the vector-reservoir contact, but it is neither expected nor has been demonstrated to have any effect on the reduction of the vector population.

*Indoor residual spraying (IRS)* - The main strategy of chemical vector control is based on the use of insecticides, with residual action as the main step in the collective protection. There are several classes of insecticides with residual action: organochlorines (DDT), organophosphates (malathion), carbamates and synthetic pyrethroids (cypermethrin, deltamethrin, among others) (WHO 2010, MS/SVS/DVE 2014).

Among the methods used to control sandfly vectors at the beginning of the 2000s,Alexander and Maroli (2003) considered (i) the IRS of houses and animal shelters and (ii) insecticide mosquito nets (ITNs), curtains or bednets, as the most useful and feasible methods to be applied on a large scale. IRS, where each house and animal shelter are treated, was thought to be more effective in urban situations rather than in rural areas, where dwellings are widely dispersed, so only a small proportion of the total sandfly population would be controlled.

In countries where this method has been used in sustained Malaria Control Programs, as in Brazil and Venezuela, IRS is generally well accepted by householders because of the additional protection against other vectors and annoying arthropods.

Brazil is the only country in Latin America that in 1953 implemented a campaign against visceral leishmaniasis. This campaign was based on the treatment of patients, sacrifice of sick dogs and residual spraying of DDT as an emergency measure to control the vector *Lu. longipalpis *(Monteiro et al. 1994). In 1955, Deane et al. carried out the first evaluation of the impact of this insecticide, indoors and outdoors, in four localities of the state of Ceará (CE), Brazil. It was observed that the density of this sandfly greatly decreased indoors in the two treated localities compared with the two untreated. This decline was maintained until the 3rd month and also in animal shelters (sheds, corrals and pens), while the sandfly abundance in captures from donkeys were very irregular, indicating that there was no impact of this compound on the *Lu. longipalpis* population in the open air.

However, due to high operational costs, the use of IRS remained quite restricted and in 1964 all control actions were interrupted. The campaign re-started in 1980 when the report of a large number of cases began to worry health officials (Monteiro et al. 1994).

Information based on the reported experiences of professionals from Brazilian Health Departments show some disparities; there are municipalities where the actions are considered to be positive, so the strategies recommended by the Brazilian Programme Control are effective, but in others, the experience seems to be negative. Given this evidence, some variables can be considered: professional capacity and operational, biological and social resources.

As previously mentioned, it is possible that variables related to human/operational resources will constitute a critical point. In the context of vector control, a set of variables related to operational questions must be habitually assessed and some reported experiences suggest as the biggest bottleneck the lack of qualified and suitable workers (human resource). Undoubtedly, in many situations the procedures are not adequately monitored.

From the point of view of society, another point to be considered is the culture of popular groups; communities that are not informed about the diseases, to which they are usually exposed, have resistance or even refuse to accept practices that compromise their daily conditions of life.

On the other hand, because of the complaint of ecologists worldwide about environmental contamination by the indiscriminate use of DDT and because of human health hazards, attention was given to synthetic pyrethroids. One of the first field trials of these compounds was by Le Pont et al. (1989) at Paranani, a sub-Andean village of Yungas, Bolivia, where houses were sprayed at the beginning of the rainy season (January 1987) inside and outside with deltamethrin (25 mg/m^2^). Kennels, hen-houses and stacks of adobe were also treated in the same way. Sandfly captures previous to the intervention were compared with those after. As a result of the treatment, *Lu. longipalpis *disappeared from the houses and animal shelters after nine and 10 months, respectively.

Conversely, a limited reduction (2-4 weeks) in the population of *Lu. longipalpis* in the rural municipality of Santa Rita, Paraíba (PB), Brazil, was obtained by Marcondes and Nascimento (1993) in houses sprayed with the same product (deltamethrin: K-othrine CE) at 50 mg/m^2^, 25 mg/m^2 ^and 12.5 mg/m^2^, in comparison with those collected in control houses sprayed with kerosene in water (1:100), with extreme irregularity in the captures.

Cypermethrin pyrethroid 31.2% wettable powder (WP) (125 mg a.i./m^2^) was applied twice a year onto the internal and external walls of 10 houses, including appended rooms and animal shelters (chicken cops, pigsty, cow shed etc.) in Caxitu, municipality of Conde, on the south coast of PB. The results showed that the insecticide significantly reduced the indoor *Lu. longipalpis*populations during the two months after the spraying and then they started to recover. The rates of mortality were variable depending on the kind of wall, but the residual effect after the fourth month was similar for the three types of walls in the area. No effect of the insecticide spraying on the occurrence of *Lu. longipalpis *was observed in the peridomestic habitat (De Silans et al. 1998).

In the Margarita Island, the residual spraying of internal and external walls with lambda-cyhalothrin emulsifiable concentrate (EC) at 25 mg/m^2^ and spatial fogging (SF) of fenitrothion around the houses at 30 g/ha was applied in three cycles in the village Santa Ana. The densities of the indoor *Lu. longipalpis*were significantly reduced in the target locality in comparison with the densities in the control village Las Cabreras, while no significant reduction was achieved outdoors with SF. Wall bioassays showed that the residual effect of the insecticide lasted for approximately three months. It was concluded that SF should only be used in epidemic situations, while by moderately increasing the dose of the insecticide in IRS, the indoor population of *Lu. longipalpis *might be kept controlled, therefore also reducing the indoor transmission (Feliciangeli et al. 2003).

Santini et al. (2010) used deltamethrin (EC 10% w/v) in Posadas, at a dose of 25 mg/m^2^ only on the external walls of three houses, peridomestic dwellings and tree trunks up to 200 m from the houses. Three houses without spraying (control) and three houses not sprayed located just at the border of the intervention area (control of dispersion due to the insecticide) were also monitored by Centers for Disease Control and Prevention (CDC) traps for*Lu. longipalpis* abundance. The results showed a significant reduction in the sandfly captures up to seven days post-intervention in the target group, but, due to the high deviation of the data, it was not considered significantly different from the controls. Observations on human behaviour, resting outdoors during the summer at the hours of major peak of *Lu. longipalpis*females (09:30 pm-00:30 am) and *Lu. longipalpis* behaviour (e.g., landing on humans in the proximity of domestic animals) strengthened the need for a multidisciplinary approach for prevention strategies based both on biological and anthropological studies.

Barata et al. (2011) employed IRS with cypermethrin pyrethroid (125 mg/m^2^) in 10 districts of Montes Claros in the northern state of Minas Gerais, Brazil, in two cycles: November/2005 and May/2006. The insecticide was applied to the internal and external walls of 10 houses (1 per district) selected for the trial and their annexes (chicken coops, stables and warehouses) and in all neighbourhood houses. Two traps were placed in each residence for three consecutive days a month, one inside the house and another in the peridomicile, totalling 20 traps. *Lu. longipalpis* was the predominant species, of which 85.8% of the total was collected outdoors. The two months prior to each spraying campaign (September-October/2005 and March-April/2006) were compared with the subsequent periods. The results showed that, two months after spraying, a significant reduction occurred only outdoors. In the second spraying period, the differences between the pre and post-spraying were significant at two months and four months after spraying, with the residual effect lasting from two-four months.

To conclude, it is worth noting that, regardless of the formulation used (EC or WP), in all of the trials mentioned above, it was confirmed that the residual effect of pyrethroids does not exceed four months (Table).

**TABLE:** Summary of data of chemical interventions to control adult*Lutzomyia longipalpis* in foci of *American visceral leishmaniasis*

Author	Country	Insecticide	Intervention	Commercial formulation	a.i./m^2^	Intervention cycles/year	Residual power	Results indoors	Results outdoors
Deane et al. (1955)	Brazil	DDT	IRS (i, o)	No data	2 g/m^2^	2	NI	Significant reduction of *Lu. longipalpis*	Significant reduction of *Lu. longipalpis*
Le Pont et al. (1989)	Bolivia	Deltamethrin	IRS (i, o)	EC	25 mg/m^2^	1	NI	No* Lu. longipalpis* up to 9 months	No *Lu. longipalpis* up to 10 months
Marcondes and Nascimento (1993)	Brazil	Deltamethrin	IRS	EC	12.5-50 mg/m^2^	2	NI	Significant reduction of *Lu. longipalpis*up to 2 months	NA
Kelly et al. (1997)	Brazil	λ-cyhalothrin	IRS (o, a)	ME	20 mg/m^2^	NA	NI	NA	Focal coverage: 90% reduction of *Lu. longipalpis;* blanket coverage 45% reduction
De Silans et al. (1998)	Brazil	Cypermethrin	IRS (i, o)	WP	125 mg/m^2^	2	4 months	Significant reduction of *Lu. longipalpis* up to 2 months	No reduction of *Lu. longipalpis*
Feliciangeli et al. (2003)	Venezuela	λ-cyhalothrin	IRS (i, o)	EC	25 mg/m^2^	3	3 months	Significant reduction of *Lu. longipalpis*during one year of follow-up	Significant reduction of *Lu. longipalpis*
Feliciangeli et al. (2003)	Venezuela	Fenitrothion	SF (a)	ULV	30 g/ha	3	NA	NA	
Santini et al. (2010)	Argentina	λ-cyhalothrin	IRS (o, a)	EC	25 mg/m^2^	1	NI	NA	Significant reduction of* Lu. longipalpis* up to 7 days (observations up to 14 days)
Barata et al. (2011)	Brazil	Cypermethrin	IRS (i, o, a)	No data	125 mg/m^2^	2	NI	Reduction of *Lu. longipalpis * up to 4 months	Reduction of *Lu. longipalpi*s up to 4 months
Courtenay et al. (2007)	Brazil	Deltamethrin	ITNs	EC	25 mg/m^2^	NA	NI	Significant reduction of human landing rate and mortality	NA
Feliciangeli et al. (2011)	Venezuela	Deltamethrin	LLNs	No data	55 mg/m^2^	NA	Good up to 10 months (time of observation)	No significant reduction of* Lu. longipalpis*	NA
MS/SVS/DVE (2014)	Brazil	Cypermethrin	(i, o, a)	WP 20, WP 30, WP 31, WP 25, WP 40	125 mg/m^2^	2	3 months	-	-

a.i.: active ingredient; a: annexes; EC: emulsifiable concentrate; i: indoors; IRS: indoor residual spraying*; *ITN: insecticide mosquito net; LLN: long lasting net; ME: microencapsulated; NA: not applicable; NI: no information; o: outdoor; SF: spatial fogging; ULV: ultra low volume; WP: wettable poder.

On the other hand, it is worth remembering that *Lu. longipalpis* is a "species complex" whose populations could have different responses to the actions of insecticides, including vectorial competence (Arrivillaga & Feliciangeli 2001, Arrivillaga et al. 2002, Bauzer et al. 2007, Arrivillaga & Marrero 2009) and that the susceptibility of sandflies to the insecticide spectrum is still poorly known (WHO 2010).

From this view, the development of monitoring networks for evaluating insecticide resistance in populations of *Lu. longipalpis* could provide important evidence contributing to the better understanding of this process. On this basis, after analysing the "pros and cons", Marcondes and Costa (2013) recently launched a very restrained proposal about the return to the use of DDT for the control of *Lu. longipalpis*, mainly because of its long residual action*.*They argued that, with the aim to save lives, carefully designed evaluations of DDT's efficacy compared with other insecticides and dog culling, should be urgently funded and developed. However, due to the opposite position of environmentalists and also the existence of regulatory laws that govern the use of this insecticide in several countries, this product should not appear as an option in public policies to control the vector of AVL (WHO 2010).

The peculiarities of the chemical control of the vectors of AVL in urban areas make this a difficult and laborious action and the results have not always proved satisfactory. Therefore, other measures offering continued results with the participation of the community are seen as alternative tools. The adoption of a proposal for integrated actions emerges as a promising possibility to interfere in the dynamic populations of sandflies. According to the World Health Organization (WHO 2010), although in special circumstances some actions such as a chemical control can produce a visible effect on sandfly populations, it would be recommended that the control vectors include more than one methodology as an action-integrated control programme. Obviously, in this context, it is extremely important to understand the local epidemiology and know the appropriate aspects of vector biology (habits and habitats), food preferences and seasonality.

*ITNs and long-lasting insecticidal nets (LLINs)* - It was thought that ITNs, a cheaper method than IRS, might represent the most suitable and sustainable method of reducing intradomiciliary transmission (Alexander & Maroli 2003), as it was for malaria vectors (Curtis & Davies 2001). However, the main disadvantage of ITNs is that their discontinuous use may affect their impact on indoors vector population.

In Amazon households of the community of Pingo d'Agua, Marajó Island, state of Pará Brazil, Courtenay et al. (2007) documented the protection by ITNs vs. *Lu. longipalpis *under controlled conditions. Compared with untreated nets, the insecticide increased the barrier effect of nets by 39%, reduced human landing rates by 80% and increased the 24-h mortality rate from 0-98% inside ITNs. Additional observations were made on the habits of the people living in that focus. They observed that people used to have dinner outdoors in the early evening and concluded that a potential predominant limiting factor for ITN efficacy in that region was a social one: bedtimes were late relative to the peak sandfly activity times. It was therefore warned that the acceptance and benefit of this measure would depend upon a good health educational program at the primary health care level that was coupled with the free provision of nets or social marketing.

The same conclusion was reached after a field trial in San Mateo, state of Aragua, Venezuela, where the obtained results showed a reduction of the sandfly density in homes with LLINs pre-treated with deltamethrin (55 mg/m^2^) (PermaNet^®^ 2.0). However, this was not significant in comparison to controls (number of sandflies collected in homes with untreated nets as well as in homes with no nets), which led to the thought that the use of the bednets was not regular, probably because the knowledge aimed at the community before implementing this control measure was not entirely appropriate (Feliciangeli et al. 2011). To test the residual power of the insecticide in treated nets, bioassays were conducted six, eight and 10 months later using*L. pseudolongipalpis *from a closed colony. The percentage of sandfly mortality in four selected points of the bednets was in between 43-98% (K Flores, unpublished observations).

*Interventions on resting sites* - In their review on *Lu. longipalpis* and the eco-epidemiology of AVL, Lainson and Rangel (2005) stressed that *Lu. longipalpis* is primordially a sylvatic species and documented its passage from the sylvatic habitat to the peridomicile, with a subsequent infestation of chicken houses and other animal shelters. In a recent study across several habitats in a metropolitan landscape, *Lu. longipalpis* was confirmed as adapted to anthropic environments. It was the most abundant species in the house and poultry yard (Pinheiro et al. 2013).

With the aim to investigate the effect of insecticides on the abundance and distribution of peridomestic *Lu. longipalpis,* Kelly et al. (1997) tested a differential application of lambda-cyhalothrin at 20 mg. a.i./m^2 ^(ICON^®^ 10% ME) in a rural area in the district of Salvatierra, Marajó Island. The interventions were: (i) the spraying of all animal pens (blanket coverage), (ii) treating a subset of animal pens, using the same method, or (iii) by the installation of lambda-cyhalothrin-impregnated 1 m^2^ cotton sheets as "targets" (focal coverage). Following blanket intervention, catches at untreated dining huts increased, possibly because the blanket coverage diverted *Lu. longipalpis* away from the major aggregation sites at animal pens and so the reduction in the abundance of *Lu. longipalpis* in CDC traps was not significant. Conversely, a 90% reduction in the *Lu. longipalpis*abundance of the sprayed sheds of the focal intervention was detected. This differential impact was attributed to the disruption of male pheromone production. It was recommended to treat all potential aggregation sites to avoid an increase in the biting rate on dogs and humans and to apply an integrated control based on: (i) treatment of houses and dining-huts with an excito-repellent insecticide, (ii) children sleeping under insecticide-treated bednets and (iii) dogs being protected by the use of some sandfly repellent, like collars.

The burden of urban AVL in Brazil led Alexander et al. (2002) to analyse the role of the domestic chicken in such new epidemiological scenario in: (i) the maintenance of the sandfly population, (ii) its attraction of reservoirs of *L. infantum* and (iii) their zooprophylactic effect. Environmental, physiologic, socioeconomic and cultural factors related to raising chickens in urban areas that might affect the transmission of *L. infantum *and whether this practice affects the risk to acquire AVL in Brazilian cities were discussed. It was suggested that the perspective would be to explore, through socioeconomic surveys, the feasibility to apply inexpensive and sustainable preventive measures that are based on the communities' willingness to participate in the control program, e.g., in the removal of chicken houses.

A new perspective arose from synthetic pheromones that are attractive to both sexes of *Lu. longipalpis* and could be used to improve the effectiveness of residual insecticide attracting sandflies to insecticide-treated animal houses, as it was tested in experimental chicken sheds (Bray et al. 2010, 2014).

*Interventions at breeding sites* - Because little is known about the breeding sites of sandflies (Feliciangeli 2004), information from laboratory and field trials to control the immature stages of* Lu. longipalpis* is lacking.

Under laboratory conditions, Warburg (1991)exposed the larvae of *Lu. longipalpis* to *Beauveria bassiana* spores smeared on a filter paper, inducing 100% mortality on day 4. However, a field assay by Reithinger et al. (1997) using a commercial product in a coffee plantation did not kill the*Lutzomyia* spp in the area. Quesada and Montoya-Lerma (1994) evaluated the effectiveness of chlorfluazuron, an insect growth-inhibitor, against *Lu. longipalpis*. Adverse effects were observed in 2nd and 3rd instar larvae as well as in adults that had ingested the insecticide at the larval stages. However, these experiments were not followed by field trials.

Deane and Deane (1957), in CE, first reported the finding of immature stages of *Lu. longipalpis* in animal corrals. However, so far, chicken houses were considered to attract both blood-seeking females and males seeking mates, but did not appear to act as breeding sites (Alexander et al. 2002). Nevertheless, very recently, an intensive search in the urban and periurban areas of two municipalities, Promissão and Dracena, endemic for AVL in SP, has provided evidence with consistent results supporting their important role as breeding sites, opening new prospects for the control of *Lu. longipalpis* at the immature stages (Casanova et al. 2013).

*Prevention methods* - *Personal protection* - In the rural and urban foci of AVL transmission, small children may be protected at night from *Lu. longipalpis* bites by wearing clothes covering their arms and legs. For people who work in the field early in the morning who cannot acquire the chemical repellents used for this purpose, a similar measure might protect them. No research on the evaluation of these activities has been reported. Alexander et al. (1995) tested the repellency and insecticidal efficacy of Nopikex™, a cheap and practical soap formulation containing 20% diethyl toluamide and 0.5% permethrin against a laboratory colony of*Lu. longipalpis* in Colombia*.* However, based on calculations of the coefficient of protection, it was observed that 8 h after the use of the soap, the repellency decreased significantly, to 67% of the initial value and no significant mortality was seen in sandflies within 24 h of exposure to the soap in the laboratory. Moreover, the repellency was lost if the soap was rinsed off the skin.

Chemical repellents are mainly recommended in foci of sylvatic transmission when people venture into the forest for business, tourism or in war situations. N, N-diethyl 3-methylbenzamide (DEET) is usually the product of choice and has been used world-wide for over 50 years (Alexander & Maroli 2003). More recently, 1-del ridinecarboxylic acid, 2-(2-hydroxyethyl)-I-methyl-propylester (KBR 3) has proven to provide individual protection against* Phlebotomus duboscqui *for 7-8 h (Perrotey et al. 2002) and it might work against *Lu. longipalpis.*

*Environmental management* - Environmental management aims to reduce man-vector contact through interventions in an ecological niche where the epidemiological chain has been occurring. Essential measures such as street cleaning, removal and disposal of organic solid waste, eliminating sources of moisture and removing domestic animals outside houses and animals shelters are procedures that can change the conditions of the environment or eliminate substrates that favour the establishment of the breeding of immature stages of vector, which impacts the population curve. Environmental management appears to be an effective tool for controlling AVL, considering that the expansion process of the disease is mainly associated with the presence and distribution of the vector *Lu. longipalpis*, especially with its ability to easily adapt to domestic habitats and its remarkable feeding plasticity (Deane 1956, Brazil & Brazil 2003, Lainson & Rangel 2005,Camargo-Neves et al. 2007, Afonso et al. 2012). The presence of sandfly populations is established when it reaches equilibrium after some time, due to a biological support: the availability of organic matter with moisture and the availability of food and shelter.

Keeping the backyard clean of trash and organic material in a state of decomposition and situating animal shelters as far as possible from the dwellings is a measure that can help to make the peridomicile unsuitable to the immature stages of*Lu. longipalpis*. In the urban focus of Posadas, Santini et al. (2010) recommended that pets and domestic animals should be kept at least 5 m from humans as it was observed that*Lu*.* longipalpis *did not land on man at 5 m and 3 m from the dog, although the CDC traps collected 140 and 228 individuals, respectively, on the same nights.

The relocation of human settlements away from sandfly habitats (WHO 2010) might be considered as an alternative measure where dwellings are close to or in the woodland. This situation has been demonstrated as a risk factor for the prevalence of infection of *Leishmania* spp in a rural AVL focus in Venezuela. The prevalence was in fact consistently associated with the *Lu. pseudolongipalpis* abundance and this was negatively correlated with the distance from the woodland (Feliciangeli et al. 2006). Similarly, in the urban foci of Teresina, the high incidence of AVL was associated with peripheral neighbourhoods with heaviest vegetation cover, subject to rapid occupation and a lack of adequate sanitary infrastructures (Cerbino Neto et al. 2009).

*Health education and social-based strategies* - Several manuals for the prevention and control of AVL in Latin American countries are available online and they all include health education as an important component of control programs. Health education is a core element in the implementation of any prevention and in the control of diseases (WHO 2010) and it is the key to controlling AVL (Killick-Kendrick 2010).

Knowledge about the disease from the political level to the communities is essential for an adequate approach to the prevention and control of this disease. Political willingness and commitment as well as intersectorial cooperation between ministries and agencies are crucial to guarantee the success of the control actions (WHO 2010).

In this context, the approach of addressing the problem through a transdisciplinary team in which experts and representatives of affected populations might share experiences and wisdom arises as a promising perspective. In this direction, studies are needed about the knowledge, attitudes and practices (KAP) of the populations at risk. Additionally, this tool may be useful to evaluate the impact of a control program (García & Borges 2010).

The creation of a healthy environment, focusing on eliminating or reducing the transmission of AVL, depends upon practices that involve the community in a partnership with health services. Therefore, community participation in the control of *Lu. longipalpis* involves an educational process that aims to encourage such participation in a way that enhances and integrates popular knowledge in their practices.

Health education allows for the formation of deeply grounded notions about disease transmission, the filling in of gaps in the understanding of their habits and knowledge of the preferred places to live in conditions favourable to its development; this new knowledge can contribute to prevention and control. We must consider the importance of planning a popular education health programme given information that is based on education and communication activities at the local level, where community participation is essential to the implementation and success of such a program. Personal protective measures should also be provided to communities, such as use of fine mesh in windows and musketeers (MS/SVS/DVE 2014). Considering that *Lu. longipalpis* has crepuscular and nocturnal activity (Forattini 1973, Brazil & Brazil 2003), human activities in places where the vector can be found should be avoided during these periods.

It is recommended that some education and health activities are included in all services that develop control actions for AVL vectors, considering the following strategies: (i) divulging to the community information about the occurrence of the disease in the region, with warnings about the signs and clinical services for diagnosis and treatment, and (ii) training teams and developing practices in the community, through health and education services, with a wide spread of strategies and educational materials informing about the disease and its transmission, in accordance with the attitudes, practices and living conditions of the local groups (MS/SVS/DVE 2014).

In Brazil, a KAP study was conducted in the endemic areas of MA amongst the rural population of the Codó township and in peripheral urban areas (an old settlement, Maracanã, on the outskirts of, with old establishment and the occurrence of AVL) and Vila Nova/Bom Viver, Paço do Lumiar township (with recent establishment and occurrence of cases). Although people were in general aware of the disease and they knew the vector and the reservoir, they did not know how to control the disease. It was concluded that the knowledge about several aspects of AVL were poor in both the rural and the peripheral urban areas (Gama et al. 1998).

To evaluate the impact of the AVL control program implemented during the years from 1998-2000, in which a health education program was one of the measures applied in Venezuela, a KAP study was carried out in two communities, La Pista and La Guardia, on the Margarita Island. The main differences between the two foci were the incidence of AVL, the socioeconomic level and the time of residence in the endemic area. Through interviews, it was found that people living in La Pista, the community with longer residence in the area, knew more about the disease. However, knowledge about prevention and control was very poor in both localities. It was stressed that KAP studies previous to the control actions are necessary to obtain a baseline for the management and sustainability of educational programs about the prevention and control of VL (García & Borges 2010).

Furthermore, KAP studies are required as baseline data or input for the design of intervention strategies, as health education programs should not be isolated or "top down" unidirectional measures. Research on the social determination of AVL risk and the actual agency of the individuals to accomplish the heath system recommendations, in addition to the social construction and perception of risk by each social group involved, also contribute to contextualising the problem and the solutions. Therefore, the transdisciplinary systemic perspective, joining the biological and social contextualisation, is an essential approach to promote community participation beyond just community collaboration. This participation implies thinking together with the community about the more appropriate measures for environment and animal management and personal protection as applied in the actual territory where the people are living. Multisectorial involvement is also necessary for sustainable programs, defining the activities and responsibilities of the public and private sectors and consolidating a common frame of risk (Salomón et al. 2012, Feliciangeli 2014).

## Concluding remarks - recommendations

The analysis of the spread of AVL and urbanisation of *Lu. longipalpis* requires us to discriminate between both issues according to spatial and temporal scales. The actual mechanism of dispersion of the vector at each scale by field and genetic research should be addressed to contribute to design control strategies.

At the macro-scale, to think in the frame of macro-economic trends, the consequent environmental/climatic changes and social determination of health would allow us to define prevention policies and control strategies at the country-continental level. In actual or potential risk areas, the main projects (railroads, dams, deforestation etc.) that imply environmental intervention and human movements should include leishmaniases in the assessment of risk and an appropriate monitoring design and responsibility definitions to act *ex-ante* eventual outbreaks.

In the meso-scale, to understand the factors that drive the deforestation-urbanisation process, the distribution and the metapopulation dynamics of the urban *Lu. longipalpis* will improve the resource allocation and prevention measures performance at the programmatic and county level. The local environmental disturbances and changes in land use with the growth of peripheral neighbourhoods (deforestation) and disorganised urbanisation with poor sociosanitary conditions (land value and social determination) should also be monitored for leishmaniasis risk.

At the micro-scale, the factors that determine the presence and abundance of the vectors are better known, allowing us to work together with the community and the county to design sustainable control strategies adapted to the local level and focused on the private and public environments, domestic animal management and the actual capacity of agency of the families to afford the programmatic recommendations.

The current knowledge about the control of *Lu. longipalpis* indicates that it is required to be part of an integrated control program that involves transdisciplinary teams at least at the country level, with experts from the bio-medical, bio-ecological and social sciences in the frame of the ecohealth systemic perspective. Such a program would thus include chemical focus control, environmental management, health education and community participation. This program should include the periodic training of agents of health, zoonosis control, education and environmental protection agencies (WHO 2010, Salomón et al. 2012,PAHO 2013, Feliciangeli 2014, MS/SVS/DVE 2014).

The different eco-socioepidemiologic contexts of each endemic area and focus make impractical any standard protocol for control. Furthermore, there are gaps in the knowledge related to AVL transmission in many regions. However, to discuss control measures, two main scenarios in America should be discriminated: rural AVL and urban AVL. On the other hand, "first case" studies require specific designs to assess autochthonous transmission.

Rural AVL usually includes old foci of low and stable endemicity due to the equilibrium between the components of the epidemiological cycle. It should be stressed that this equilibrium could be broken by human interventions in the environment or unusual climate events at any scale. Focused on vector control, the inputs to characterise this epidemiological scenario should include ecologic information of the area together with social studies (i.e., KAP surveys, in-depth interviews, key informants, work practices-related risk) and entomological studies (i.e., seasonality, endophagy, place-hours of domestic-peridomestic-extradomestic activity). The control program agents could perform these studies together with researchers by intersectoral collaboration. The analysed information, when shared and discussed with decision-makers, relevant stakeholders and the involved community, will allow us to translate the research results into actual, feasible and effective measures (when, where, how) and the contents of health education programs for empowering the actors. In this sense, health promoters and community leaders could follow and evaluate the proposed measures, providing further sustainability. The use of LLINs is recommended, especially in youngsters, but it requires prior work to solve issues about acceptability, affordability, equity and correct use. On the other hand, entomological surveillance at the local level in a rural focus of low steady endemicity should be performed as a "continuous observation and assessment of information" (Gomes 2002) with qualitative and quantitative goals (MS/SVS/DVE 2014), mainly to make early warnings about a change in transmission rates, although unfortunately the rise of cases is usually the first alert. Expected changes in the economic and demographic trends, environment and work structure should generate intensified surveillance at the appropriate scale. An actual or eventual rise in vectors, vector-human effective contacts or parasite circulation could be diminished by IRS, usually an acceptable strategy for rural householders.

Urban AVL could appear as an outbreak due to the migration of infected humans and dogs from endemic areas and then progress to hyperendemic or steady to low epidemic foci. The vector control design requires the same social and entomological research inputs as an rural AVL study, but includes some particular issues such as the stratification of risk within the city (Geographic Information Systems - "hot spots" in space and time). Social studies should be aware of the social determination and consequent spatial segregation of risk as well as the different accessibility to the health system and basic urban sanitary services between different social groups. The practices and actors associated with the transit, traffic and management of dogs should be taken into account, as ownership practices have individual, corporative (veterinarians, breeders, animal welfare nongovernmental organisations) and public sector (dog population programs and public areas) responsibilities. Therefore, the intersectoral teams in urban scenarios have a broader composition, including the media and agents from different county services (waste management, development). Regarding programmatic surveillance, it should be designed according to the intensity of transmission in the locality and the space-time distribution of vector abundance inside the locality. Due to the lack of stronger evidence for residual effectivity, chemical control with IRS is not a generally recommended measure in urban foci. Additionally, indoor insecticide cycles could be rejected by urban householders. Otherwise, IRS requires eco-epidemiological scenarios that are fairly characterised and must be performed with a supervised protocol of application and a rigorous design of evaluation (pre-intervention baseline, post-intervention impact on vector population and AVL transmission). Furthermore, the operational research on AVL and *Lu. longipalpis* control should require standardised protocols for the impact assessment of integrated actions, both in the short and long term. Networks are also needed for monitoring resistance to insecticide in wild and domestic populations of *Lu. longipalpis*, in addition to studies on sibling and other species vectorial capacity, such as *Lutzomyia cruzi* (de Pita-Pereira et al. 2008, Brazil et al. 2010) and*Migonemyia migonei *(Carvalho et al. 2010).
